# Changes in the characteristics and outcomes of COVID-19 patients from the early pandemic to the delta variant epidemic: a nationwide population-based study

**DOI:** 10.1080/22221751.2022.2155250

**Published:** 2022-12-20

**Authors:** Koichi Miyashita, Hironao Hozumi, Kazuki Furuhashi, Eiji Nakatani, Yusuke Inoue, Hideki Yasui, Masato Karayama, Yuzo Suzuki, Tomoyuki Fujisawa, Noriyuki Enomoto, Naoki Inui, Toshiyuki Ojima, Takafumi Suda

**Affiliations:** a Second Division, Department of Internal Medicine, Hamamatsu University School of Medicine, Hamamatsu, Japan; b Graduate School of Public Health, Shizuoka Graduate University of Public Health, Shizuoka, Japan; c Department of Clinical Pharmacology and Therapeutics, Hamamatsu University School of Medicine, Hamamatsu, Japan; d Department of Community Health and Preventive Medicine, Hamamatsu University School of Medicine, Hamamatsu, Japan

**Keywords:** COVID-19, epidemiology, risk factors, national database of health insurance claims and specific health checkups of Japan, NDB

## Abstract

The coronavirus disease 2019 (COVID-19) pandemic has dramatically changed because of virus mutations, vaccine dissemination, treatment development and policies, among other factors. These factors have a dynamic and complex effect on the characteristics and outcomes of patients. Therefore, there is an urgent need to understand those changes and update the evidence. We used a large-scale real-world data set of 937,758 patients with COVID-19 from a nationwide claims database that included outpatients and inpatients in Japan to investigate the changes in their characteristics, outcomes and risk factors for severity/mortality from the early pandemic to the delta variant-predominant waves. The severity of COVID-19 was defined according to the modified World Health Organization clinical-progression ordinal scale. With changing waves, mean patient age decreased, and proportion of patients with comorbidities decreased. The incidences of “severe COVID-19 or death (i.e. ≥severe COVID-19)” and “death” markedly declined (5.0% and 2.9%, wild-type-predominant; 4.6% and 2.2%, alpha variant-predominant and 1.4% and 0.4%, delta variant-predominant waves, respectively). Across the wave shift, risk factors for ≥ severe COVID-19 and death, including older age, male, malignancy, congestive heart failure and chronic obstructive pulmonary disease, were largely consistent. The significance of some factors, such as liver disease, varied as per the wave. This study, one of the largest population-based studies on COVID-19, showed that patient characteristics and outcomes changed during the waves. Risk factors for severity/mortality were similar across all waves, but some factors were inconsistent. These data suggest that the clinical status of COVID-19 will change further with the coming epidemic wave.

## Introduction

Severe acute respiratory syndrome coronavirus 2 that causes coronavirus disease 2019 (COVID-19) began to cause alarm at the end of 2019, spread worldwide and caused a significant disease burden on the international community. Since the World Health Organization (WHO) declared a pandemic on 11 March 2020 until the end of May 2022, > 530 million people worldwide have been affected by the disease, and approximately 6 million people have died [1]. During this time, the virus has mutated and many variants have emerged, of which the WHO has designated five variants (alpha, beta, gamma, delta and omicron) as variants of concern (VOCs) [2]. Although treatments for COVID-19 are being established and vaccination against the virus is becoming more widespread [3,4], the spread of COVID-19 has not been controlled. Therefore, there continues to be a need to update the evidence on COVID-19 and improve its management.

Epidemiological studies and identification of risk factors for increased severity/mortality are essential for improving patient management and deciding on policies for COVID-19. In particular, such risk factors are published in each country’s guidance [5,6] and are used in determining the treatment regimen and priority population for vaccination or medical care [7]. However, the evidence is based primarily on studies limited to hospitalised patients or studies conducted during the early pandemic [8–20]. Reported mortality rates vary widely across studies, ranging from 14.8% to 30.8% and from 0.28% to 24.3% in inpatient and outpatient studies, respectively. Therefore, validation or update with a larger-scale population-based study, including both inpatients and outpatients, is essential. It is also unclear if the risk factors for increased severity/mortality after the VOCs epidemic or after widespread vaccination are the same as those in the early pandemic. Collectively, in the real world, several factors, including VOCs, vaccine dissemination, treatment development and policies, have a complex effect on the characteristics and outcomes of COVID-19 patients. The National Database of Health Insurance Claims and Specific Health Checkups of Japan (NDB) is one of the largest databases in the world and covers >126 million people and 1.9 billion claims annually, including >99% of these Japanese claims data, both inpatient and outpatient [21]. This study using large-scale, real-world data from the NDB aimed to investigate changes in the characteristics, outcomes and risk factors for severity/mortality of COVID-19 patients from the early pandemic to the delta variant epidemic.

## Materials and methods

### Dataset

The NDB includes information relating to age, sex, diseases based on the International Statistical Classification of Diseases and Related Health Problems (ICD-10), prescribed drugs and medical procedures covered by insurance and mortality, but not information on vaccination. All adult patients diagnosed with COVID-19 during the study period (between January 2020 and August 2021) were included in this study and patient data at the time of the first COVID-19 diagnosis during the study period were extracted from the NDB. During this period, almost all patients with COVID-19 in Japan had a diagnosis confirmed by the nucleic acid amplification method (*e.g.* reverse-transcription polymerase chain reaction) or antigen testing. We have also extracted data regarding diseases listed in the Charlson Comorbidity Index (Supplementary Table 1) [22], which has been widely used for evaluating risk adjustment in outcome studies. Of these comorbidities, chronic pulmonary diseases were divided into asthma, chronic obstructive pulmonary disease (COPD) and other chronic pulmonary diseases. Data on patients with acquired immunodeficiency syndrome/human immunodeficiency virus are not presented owing to anonymity and statistical concerns because the number of patients was very small. For treatments against COVID-19, data on relevant drugs used ≤60 days after COVID-19 diagnosis were extracted. For respiratory supportive care, data on the use of oxygen, high-flow nasal cannula therapy (HFNC), invasive or noninvasive mechanical ventilation (MV) and extracorporeal membrane oxygenation (ECMO) ≤ 60 days from the date of COVID-19 diagnosis were extracted.

This study was conducted in accordance with the Declaration of Helsinki and approved by the institutional review board of the Hamamatsu University School of Medicine (approval number: 21–024). Written informed consent was not required owing to the retrospective nature of the study, which used a dataset anonymised by the Japan Ministry of Health, Labour and Welfare.

### Outcomes

Outcomes were classified into four categories according to the WHO clinical-progression ordinal scale with slight modifications [23]: mild, moderate, severe COVID-19 and death, based on the maximum severity ≤60 days of the COVID-19 diagnosis. Each category of disease was defined as follows: (1) mild, no oxygen supplementation, HFNC, MV or ECMO required; (2) moderate, requires oxygen supplementation but not HFNC, MV or ECMO; (3) severe, requires use of HFNC, MV or ECMO; (4) death. Patients with severe or worse outcomes, *i.e.* those who met the criteria for severe illness or death were grouped together and defined as ≥ severe COVID-19.

### Waves

The NDB does not contain information on the variants confirmed in each patient. Therefore, based on reports of the trends of variants detected in Tokyo, Japan [24], the period when the detection rate of a particular VOC exceeded 50% of the number of tests performed was defined as the predominant wave of that VOC, and the study period was divided as follows: (1) wild-type-predominant, from 1 January 2020–18 April 2021; (2) alpha-predominant, from 19 April 2021–18 July 2021 and (3) delta-predominant, from 19 July 2021–31 August 2021 (Supplementary Figure 1).

### Statistical analysis

We explored the occurrences of severe COVID-19 and death in each wave and overall as well as the corresponding associated factors. Categorical variables are expressed as number (%). To compare proportions, the risk difference and risk ratio and their corresponding 95% confidence intervals (CIs) were calculated using the Wald test-based method. Univariable and multivariable logistic regression model for ≥ severe COVID-19 and for death were used to identify risk factors, such as age, sex, wave and comorbidities, and the odds ratio (OR), 95% CI and *P*-values were also calculated. The multicollinearity between variables was checked, and variable selection in the multivariable model was not performed. In all analyses, *P* < .05 was accepted as indicating statistical significance. All data were analysed using SAS software, version 9.4 (SAS Institute Inc., NC, USA).

## Results

### Changes in the clinical characteristics, severity and outcome by wave

A total of 937,758 adult patients diagnosed with COVID-19 were identified (Table 1). Of these patients, 365,929 (39.0%), 196,957 (21.0%) and 374,872 (40.0%) were diagnosed during the wild-type-, alpha – and delta-predominant waves, respectively. As the wave shifted from the wild-type-predominant to delta-predominant, the proportion of patients aged 20–49 years increased, whereas that of patients aged ≥65 years markedly decreased (wild-type-predominant wave: 28.9%, alpha-predominant wave: 22.6% and delta-predominant wave: 6.7%), which indicated a decreasing median age range from 45–49 years in the wild-type-predominant wave to 35–39 years in the delta-predominant wave. The proportion of patients with any comorbidity decreased during the wave shift. However, male predominance remained.

**Table 1.** Patient characteristics, treatments and outcomes.

	All n = 937,758	Predominant Wave^a^		
		Wild-type *n* = 365,929	Alpha *n* = 196,957	Delta *n* = 374,872
Age, years	40–44^b^	45–49^b^	45–49^b^	35–39^b^
20–49	577,256 (61.6)	187,066 (51.1)	111,240 (56.5)	278,950 (74.4)
50–64	185,088 (19.7)	72,976 (19.9)	41,213 (20.9)	70,899 (18.9)
65–79	104,561 (11.2)	60,749 (16.6)	27,492 (14.0)	16,320 (4.4)
80 –	70,853 (7.6)	45,138 (12.3)	17,012 (8.6)	8703 (2.3)
Sex, male	511,035 (54.5)	195,235 (53.4)	106,447 (54.0)	209,353 (55.8)
Comorbidity,				
Cerebrovascular disease	54,050 (5.8)	31,838 (8.7)	12,911 (6.6)	9301 (2.5)
Any malignancy	42,854 (4.6)	24,379 (6.7)	10,062 (5.1)	8413 (2.2)
Dementia	27,823 (3.0)	17,999 (4.9)	6562 (3.3)	3262 (0.9)
Myocardial infarction	9016 (1.0)	5306 (1.5)	2148 (1.1)	1562 (0.4)
Renal disease	20,638 (2.2)	12,105 (3.3)	4938 (2.5)	3595 (1.0)
Congestive heart failure	57,775 (6.2)	33,809 (9.2)	13,716 (7.0)	10,250 (2.7)
Peripheral vascular disease	33,768 (3.6)	18,697 (5.1)	8139 (4.1)	6932 (1.8)
Chronic pulmonary disease,	119,927 (12.8)	60,531 (16.5)	25,654 (13.0)	33,742 (9.0)
Asthma	81,553 (8.7)	39,676 (10.8)	17,396 (8.8)	24,481 (6.5)
Chronic obstructive pulmonary disease	7273 (0.8)	4393 (1.2)	1653 (0.8)	1227 (0.3)
Others	55,939 (6.0)	30,433 (8.3)	11,926 (6.1)	13,580 (3.6)
Rheumatic disease	13,889 (1.5)	7294 (2.0)	3224 (1.6)	3371 (0.9)
Peptic ulcer disease	67,798 (7.2)	35,831 (9.8)	15,367 (7.8)	16,600 (4.4)
Liver disease	77,601 (8.3)	38,149 (10.4)	18,337 (9.3)	21,115 (5.6)
Diabetes mellitus	112,102 (12.0)	59,371 (16.2)	27,099 (13.8)	25,632 (6.8)
Hemiplegia or paraplegia	3714 (0.4)	2293 (0.6)	854 (0.4)	567 (0.2)
Metastatic solid tumours	3269 (0.3)	1864 (0.5)	728 (0.4)	677 (0.2)
Treatment after diagnosis of COVID-19				
Dexamethasone	110,082 (11.7)	46,048 (12.6)	33,922 (17.2)	30,112 (8.0)
Steroid pulse	12,366 (1.3)	5550 (1.5)	4026 (2.0)	2790 (0.7)
Tocilizumab	7723 (0.8)	3089 (0.8)	2526 (1.3)	2108 (0.6)
Baricitinib	11,947 (1.3)	231 (0.06)	5426 (2.8)	6290 (1.7)
Heparin	37,627 (4.0)	19,306 (5.3)	10,759 (5.5)	7562 (2.0)
Respiratory support care,				
Oxygen supplementation	101,224 (10.8)	50,932 (13.9)	28,207 (14.3)	22,085 (5.9)
High-flow nasal cannula	13,601 (1.5)	4006 (1.1)	3758 (1.9)	3076 (0.8)
Mechanical ventilation	10,840 (1.2)	8011 (2.2)	3505 (1.8)	2085 (0.6)
Extracorporeal membrane oxygenation	799 (0.09)	472 (0.13)	174 (0.09)	153 (0.04)
Outcomes				
Mild	833,407 (88.9)	312,904 (85.5)	168,037 (85.3)	352,466 (94.0)
Moderate	71,945 (7.7)	34,907 (9.5)	20,037 (10.2)	17,001 (4.5)
Severe	15,702 (1.7)	7340 (2.0)	4643 (2.4)	3719 (1.0)
Death	16,704 (1.8)	10,778 (2.9)	4240 (2.2)	1686 (0.4)

Data are presented as number (%).

^a^ Wild-type-predominant wave, 1 January 2020–18 April 2021; alpha-predominant wave, 19 April 2021–18 July 2021; delta-predominant wave, 19 July 2021–31 August 2021.

^b^ Median age category.

The proportion of patients who required any pharmacological treatment, including dexamethasone (wild-type-predominant wave: 12.6%, alpha-predominant wave: 17.2% and delta-predominant wave: 8.0%), and the proportion of those who required oxygenation (13.9%, 14.3% and 5.9%, respectively) or HFNC (1.1%, 1.9% and 0.8%, respectively) increased as the wave type shifted from the wild-type-predominant wave to the alpha-predominant wave, but those proportions decreased during the delta-predominant wave. The proportion of patients who required MV (2.2%, 1.8% and 0.6%, respectively) and ECMO (0.13%, 0.09% and 0.04%, respectively) decreased during the wave shift.

**Figure 1.**
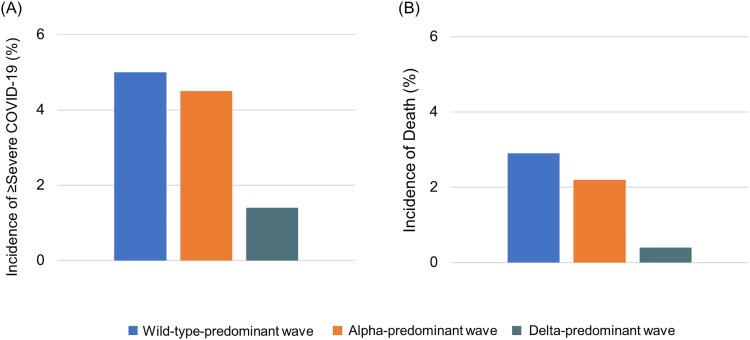
**Incidences of ≥ severe COVID-19 and of death by wave.** During the wild-type-, alpha – and delta-predominant waves, the incidence of ≥ severe COVID-19 ^a^ was 5.0%, 4.5% and 1.4%, respectively. During the wild-type-, alpha – and delta-predominant waves, the incidence of death was 2.9%, 2.2% and 0.4%, respectively. ^a^ Based on the total number of patients with an outcome of severe COVID-19 or death.

The incidence of severe outcomes or death (i.e. ≥severe COVID-19) markedly declined during the wave shift. The incidence was lower during the alpha-predominant wave (4.5%) than during the wild-type-predominant wave (5.0%) (risk difference −0.4%, 95% CI −0.6% to −0.3%) and was also lower during the delta-predominant wave (1.4%) than during the alpha-predominant wave (4.5%) (risk difference −3.1%, 95% CI −3.2% to −3.0%) (Figure 1 A and Supplementary Table 2). Additionally, the incidence of death significantly decreased over the wave shift. The incidence was lower during the alpha-predominant wave (2.2%) than during the wild-type-predominant wave (2.9%) (risk difference −0.8%, 95%CI −0.9% to −0.7%) and was much lower during the delta-predominant wave (0.4%) than during the alpha-predominant wave (2.2%) (risk difference −1.7%, 95%CI −1.8% to −1.6%) (Figure 1B and Supplementary Table 2).

### Incidences of ≥ severe COVID-19 and of death by age with waves

**Figure 2.**
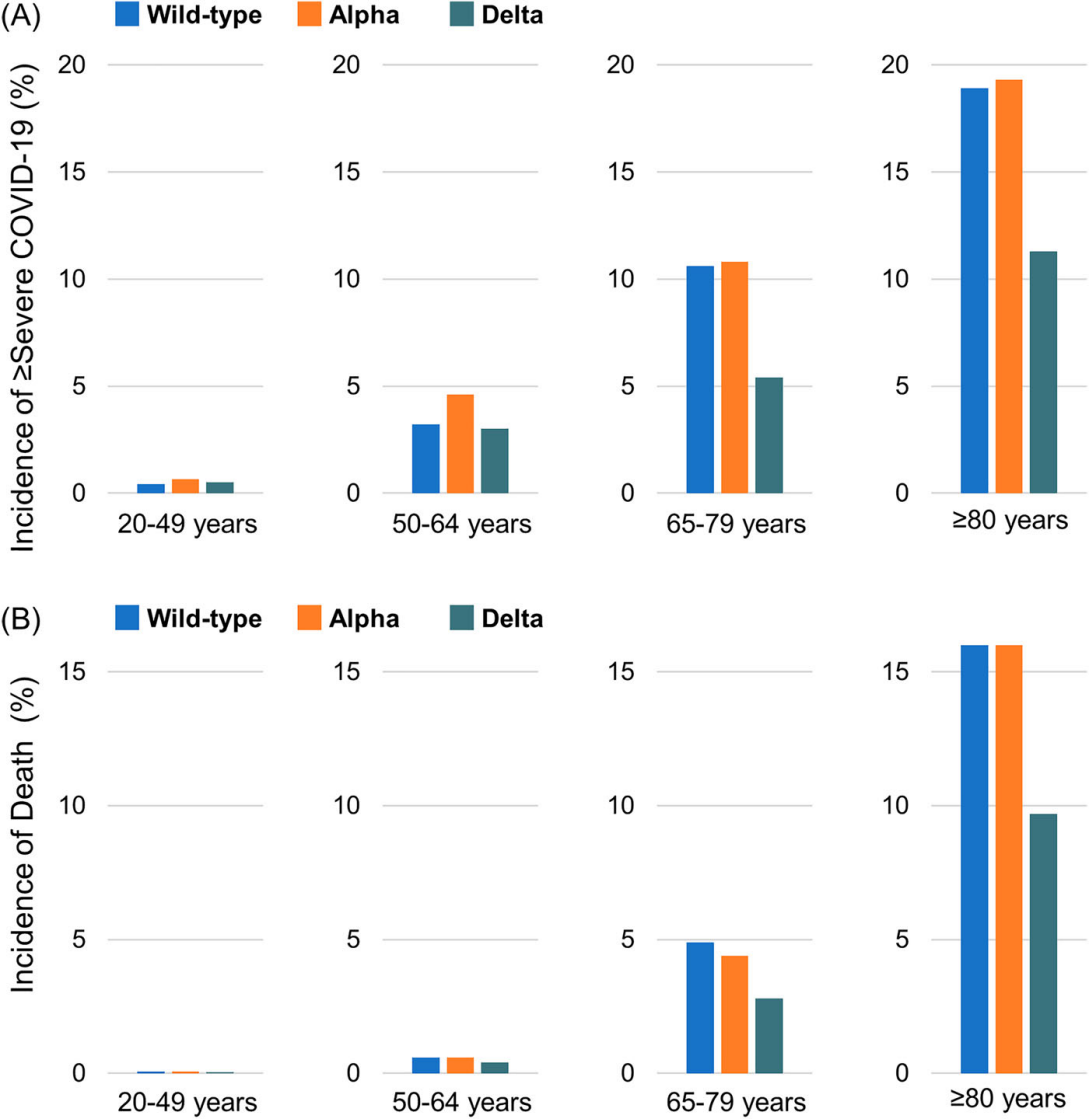
**Incidences of ≥ severe COVID-19 and of death by age group and waves.** During the wild-type-, alpha – and delta-predominant waves, the incidence of ≥ severe COVID-19 ^a^ was 0.4%, 0.7% and 0.5%, respectively, in the 20–49 years age group; 3.2%, 4.6% and 3.0%, respectively, in the 50–64 years age group; 10.6%, 10.8% and 5.4% respectively, in the 65–79 years age group and 18.9%, 19.3% and 11.3%, respectively, in the ≥80 years age group. During the wild-type-, alpha – and delta-predominant waves, the incidence of death was 0.07%, 0.06% and 0.04%, respectively, in the 20–49 years age group; 0.6%, 0.6% and 0.4%, respectively, in the 50–64 years age group; 4.9%, 4.4% and 2.8%, respectively, in the 65–79 years age group; and 16.0%, 16.0% and 9.7%, respectively, in the ≥80 years age group. ^a^ Based on the total number of patients with an outcome of severe COVID-19 or death.

Table 2 shows the risk differences and ratios in the ≥ severe COVID-19 and death groups by age and wave. In the 20–49 and 50–64 years groups (Figure 2A), the incidence of ≥ severe COVID-19 was lower during the delta-predominant wave than during the wild-type – or alpha-predominant wave, but the risk differences were small. In the 65–79 and ≥80 years groups, the incidence of ≥ severe COVID-19 during the delta-predominant wave was the lowest of the three waves. The risk differences between the delta-predominant wave and wild-type – or alpha-predominant wave were relatively larger in the 65–79 and ≥80 years groups than in the 20–49 and 50–64 years groups.

**Table 2.** Risk differences and risk ratios in ≥ severe COVID-19 and death by age group and wave.

	Wave^a^			Wild-type vs. Alpha^b^		Wild-type vs. Delta^b^		Alpha vs. Delta^b^	
	Wild-type	Alpha	Delta	Risk difference % (95%CI)	Risk ratio (95%CI)	Risk difference % (95%CI)	Risk ratio (95%CI)	Risk difference % (95%CI)	Risk ratio (95%CI)
20–49 years	*N* = 187,066	*N* = 111,240	*N* = 278,950						
≥Severe^c^	791 (0.42)	734 (0.66)	1432 (0.51)	0.24 (0.18–0.29)	1.56 (1.41–1.72)	0.09 (0.05–0.13)	1.21 (1.11–1.32)	−0.15 (−0.2 to −0.09)	0.78 (0.71–0.85)
Severe	659 (0.4)	672 (0.6)	1318 (0.5)	0.25 (0.20–0.30)	1.71 (1.54–1.91)	0.12 (0.08–0.16)	1.34 (1.22–1.47)	−0.13 (−0.18 to −0.08)	0.78 (0.71–0.86)
Death	132 (0.07)	62 (0.06)	114 (0.04)	−0.02 (−0.03–0)	0.79 (0.58–1.07)	−0.03 (−0.04 to −0.02)	0.58 (0.45–0.74)	−0.02 (−0.03–0)	0.73 (0.54–1.00)
50–64 years	N = 72,976	N = 41,213	N = 70,899						
≥Severe^c^	2344 (3.2)	1895 (4.6)	2114 (3.0)	1.4 (1.1–1.6)	1.43 (1.35–1.52)	−0.25 (−0.4 to −0.1)	0.93 (0.88–0.98)	−1.6 (−1.9 to −1.4)	0.65 (0.61–0.69)
Severe	1892 (2.6)	1638 (4.0)	1834 (2.6)	1.4 (1.2–1.6)	1.53 (1.44–1.64)	−0.01 (−0.17–0.16)	1.00 (0.94–1.06)	−1.4 (−1.6 to −1.2)	0.65 (0.61–0.69)
Death	452 (0.6)	257 (0.6)	280 (0.4)	0 (−0.1–0.1)	1.01 (0.86–1.17)	−0.2 (−0.3 to −0.2)	0.64 (0.55–0.74)	−0.2 (−0.3 to −0.1)	0.63 (0.53–0.75)
65–79 years	N = 60,749	N = 27,492	N = 16,320						
≥Severe^c^	6437 (10.6)	2979 (10.8)	875 (5.4)	0.2 (−0.2–0.7)	1.02 (0.98–1.07)	−5.2 (−5.7 to −4.8)	0.51 (0.47–0.54)	−5.5 (−6.0 to −5.0)	0.49 (0.46–0.53)
Severe	3468 (5.7)	1781 (6.5)	427 (2.6)	0.7 (0.4–1.1)	1.13 (1.07–1.20)	−3.1 (−3.4 to −2.8)	0.46 (0.42–0.51)	−3.9 (−4.2 to −3.5)	0.40 (0.36–0.45)
Death	2969 (4.9)	1198 (4.4)	448 (2.8)	−0.5 (−0.8 to −0.2)	0.89 (0.84–0.95)	−2.1 (−2.4 to −1.8)	0.56 (0.51–0.62)	−1.6 (−2.0 to −1.3)	0.63 (0.57–0.70)
80 – years	N = 45,138	N = 17,012	N = 8703						
≥Severe^c^	8546 (18.9)	3275 (19.3)	984 (11.3)	0.3 (−0.4–1.0)	1.02 (0.98–1.05)	−7.6 (−8.4 to −6.9)	0.60 (0.56–0.64)	−7.9 (−8.8 to −7.1)	0.59 (0.55–0.63)
Severe	1321 (2.9)	552 (3.2)	140 (1.6)	0.3 (0–0.6)	1.11 (1.01–1.22)	−1.3 (−1.6 to −1.0)	0.55 (0.46–0.65)	−1.6 (−2.0 to −1.3)	0.50 (0.41–0.60)
Death	7225 (16.0)	2723 (16.0)	844 (9.7)	0 (−0.6–0.6)	1.00 (0.96–1.04)	−6.3 (−7.0 to −5.6)	0.61 (0.57–0.65)	−6.3 (−7.1 to −5.5)	0.61 (0.56–0.65)

^a^ Wild-type-predominant, 1 January 2020–18 April 2021; alpha-predominant, 19 April 2021–18 July 2021; delta-predominant, 19 July 2021–31 August 2021.

^b^ Earlier wave was used as reference.

^c^ Total number of patients with an outcome of severe COVID-19 or death.

CI, confidence interval

In the 20–49 and 50–64 years groups (Figure 2B), the incidences of death were lower during the delta-predominant wave than during the wild-type – or alpha-predominant wave, but the risk differences were also small. In the 65–79 and ≥80 years groups, the incidence of death during the delta-predominant wave was the lowest among the three waves. The risk differences between the delta-predominant wave and wild-type – or alpha-predominant wave were also considerably larger in the 65–79 and ≥80 years groups than in the 20–49 and 50–64 years groups.

### Risk factors for ≥ severe COVID-19 and death

The results of univariable analysis and multivariable analysis adjusted for age, sex, wave and comorbidities for all patients during the study period are shown in Supplementary Tables 3 and 4 and Supplementary Figure 2. Older age, male, cerebrovascular disease, malignancy, dementia, renal disease, congestive heart failure, COPD, other chronic pulmonary disease, rheumatic disease, peptic ulcer, diabetes mellitus, hemiplegia and metastatic solid tumours were independent risk factors for both ≥ severe COVID-19 and death.

**Figure 3.**
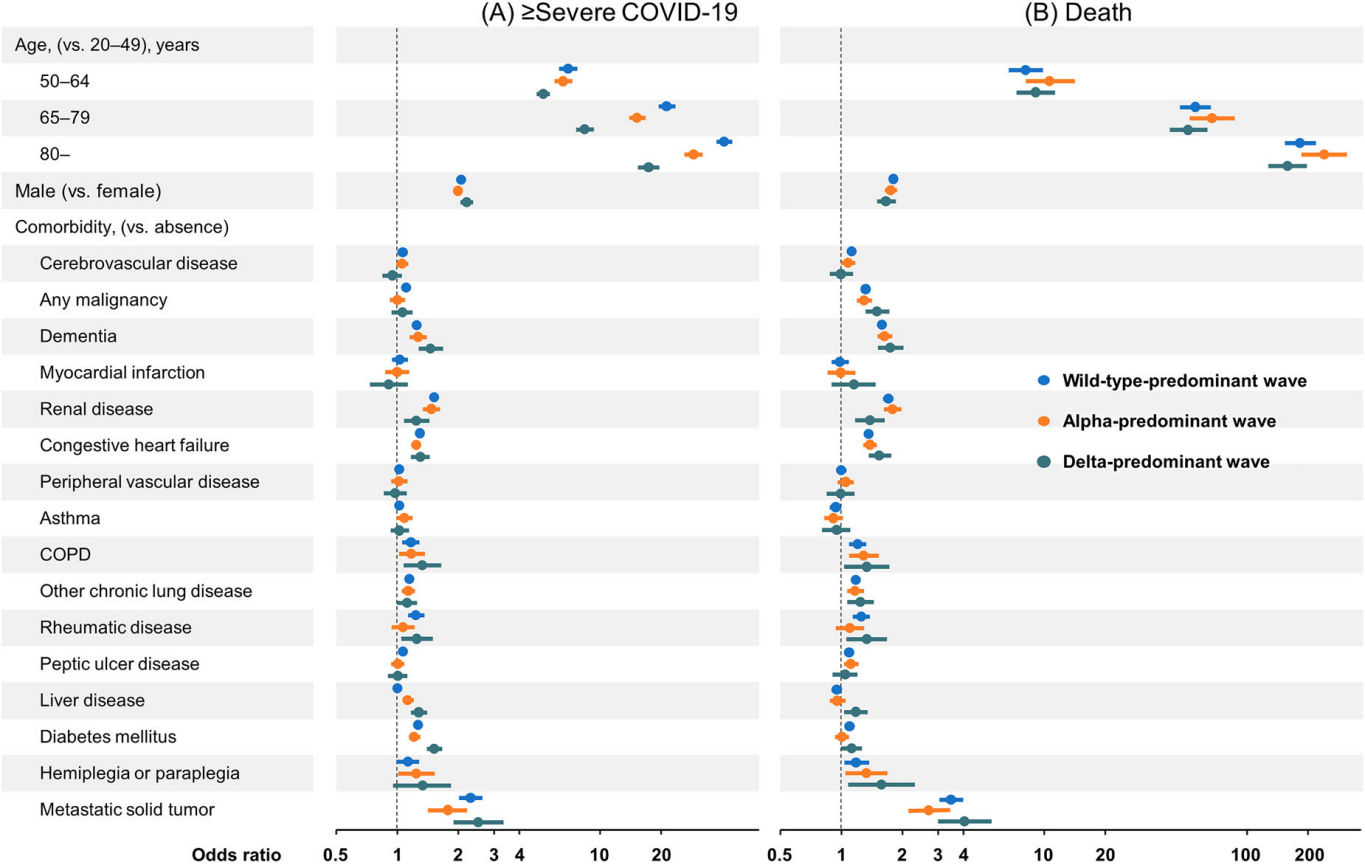
**Multivariable logistic regression analyses for ≥ severe COVID-19 and for death by wave.** Adjusted odds ratios and 95% confidence intervals were plotted. The odds ratios were adjusted for age, sex and comorbidities. Wild-type-predominant; 1 January 2020–18 April 2021, alpha-predominant; 19 April 2021–18 July 2021, delta-predominant; 19 July 2021–31 August 2021. COPD, chronic obstructive pulmonary disease.

The results of multivariable analysis adjusted for age, sex and comorbidities by wave are shown in Supplementary Tables 5 and 6 and Figure 3. For ≥ severe COVID-19, older age, male, dementia, renal disease, congestive heart failure, COPD, other chronic pulmonary disease, diabetes mellitus and metastatic solid tumours were consistent risk factors during any waves. However, peptic ulcer disease was a risk factor during the wild-type predominant wave, but not during the alpha – or delta-predominant wave. In addition, liver disease was a risk factor during the alpha – and delta-predominant waves, but not during the wild-type predominant wave. The risk for ≥ severe COVID-19 in patients with diabetes mellitus was higher during the delta-predominant wave (OR 1.53, 95%CI 1.42–1.65) than during the wild-type – (OR 1.27, 95%CI 1.22–1.31) or alpha-predominant waves (OR 1.22, 95%CI 1.15–1.28).

For death, older age, male, malignancy, dementia, renal disease, congestive heart failure, COPD, other chronic pulmonary disease, hemiplegia and metastatic solid tumours were consistent risk factors across all waves. Liver disease decreased the risk of death during the wild-type-predominant wave (OR 0.93, 95%CI 0.88–0.99), but increased the risk of death during the delta-predominant wave (OR 1.16, 95%CI 1.01–1.32).

## Discussion

This is the first and largest study to investigate changes in the characteristics, outcomes and risk factors for severity/mortality in >930,000 COVID-19 patients from the early pandemic to the delta variant epidemic. A strength of this study is that the epidemiology of patients with a confirmed diagnosis of COVID-19, including inpatients and outpatients, was analysed using a nationwide large data set covering >99% of all claims in Japan. During the delta-predominant wave, compared with the earlier waves, the median age group became younger, and the proportion of the elderly in the overall patient population decreased considerably. Accordingly, the proportion of patients having comorbidities decreased. Importantly, a marked reduction was noted in the incidences of ≥ severe COVID-19 and of death during the wave shifts, especially in the elderly. In the multivariable analysis, older age, male and particular comorbidities, such as dementia, renal disease, congestive heart failure, COPD and others, were consistent independent risk factors for ≥ severe COVID-19 and for death across the waves. However, the significance of some factors varied by waves. Liver disease was a decreased risk for death in the wild-type-predominant wave but an increased risk factor for death in the delta-predominant wave. Collectively, these observations suggest that during the wave shift of COVID-19 accompanied by virus mutations, vaccine dissemination and treatment development in the real world, there have been changes in the characteristics, severity/mortality and risk factors of COVID-19 patients.

Although the virulence of the delta variant has been reported to be higher than that of the alpha variant [25], the present study demonstrated that the incidences of ≥ severe COVID-19 and of death during the delta-predominant wave were markedly reduced by approximately 60% and 80%, respectively, compared with those during the alpha-predominant wave. There are several possible reasons for these reductions. Of these, the increased vaccination rate in Japan is thought to be the most important factor. As shown in Supplementary Figure 1, the vaccinated population in Japan had increased dramatically since the middle of the alpha-predominant wave, which led to preventing severe disease and death, even against the delta variant. Additionally, before the delta-predominant wave, treatment strategies using dexamethasone, baricitinib and remdesivir have been established [26–29], and during the delta-predominant wave, casirivimab/imdevimab became available in Japan [30–31]. Taken together, we speculated that these overall advances may have contributed to the change in the clinical status of COVID-19 patients, including significant improvement in outcomes.

In the present study, the proportion of elderly patients among all adult patients substantially decreased during the delta-predominant wave compared with the preceding waves, and the incidence of severe disease and death in elderly patients declined with a large risk difference. In Japan, priority vaccination against COVID-19 for the elderly began before the delta-predominant wave. The proportion of the elderly aged ≥ 65 years who received their second vaccination was approximately 90% at the peak of the delta-predominant wave, whereas the proportion of people aged <65 years who received their second vaccination remained <25% (Supplementary Figure 1). Thus, the difference in the proportions of vaccinated persons may have resulted in more pronounced reductions in COVID-19 infections, severe disease and death among the elderly than among the young.

In the early COVID-19 pandemic, several population-based studies reported risk factors for increased severity/mortality. In a report from the United States (n = 31,461; mortality rate: 4.1%), older age, male and comorbidities, such as cardiovascular disease, congestive heart failure, dementia, chronic pulmonary disease, liver disease, renal disease and metastatic solid tumours were risk factors for death [18]. According to data from Mexico (n = 331,298; mortality rate: 11.6%), older age, male and comorbidities, such as COPD and renal disease, were risk factors for death [19]. A study from Denmark (n = 11,122; mortality rate: 5.2%) reported that comorbidities, such as heart disease, stroke, diabetes, malignancy and liver disease, were associated with a higher risk for death [20]. In the present study (n = 937,758; mortality: 1.8%) older age, male and most Charlson comorbidities, including cerebrovascular disease, malignancy, dementia, renal disease, congestive heart failure, COPD, other chronic pulmonary disease, rheumatic disease, peptic ulcer, hemiplegia and metastatic solid tumours, were risk factors for both ≥ severe disease and death, which were basically similar to the risk factors previously reported. In addition, many of those factors were consistent across the wild-type-, alpha – and delta-predominant waves. On the other hand, the significance of liver disease, peptic ulcer disease and diabetes in severity or death varied by waves. For example, liver disease was significantly associated with an increased risk for death in the delta-predominant wave, whereas this disease was significantly associated with a decreased risk during the wild-type predominant wave. The reasons for these discrepancies are unknown, but several factors, including viral variants, vaccine dissemination and therapeutic agents, may be responsible. For proper management and policymaking of COVID-19, these observations suggest that consistent risk factors should be kept in mind in every wave, but we also need to consider the possibility that the significance of these risk factors may change as the waves shift.

Since the early COVID-19 pandemic, chronic pulmonary disease has been considered a factor that increases the risk of severity and mortality. To date, many studies have reported that patients with COPD are at higher risk of COVID-19 severity and mortality than those without COPD [19,32–35]. On the other hand, it is not yet conclusively known if asthma increases the risk of severity and mortality, but a recent meta-analysis reported that asthma was not significantly associated with the risk of hospitalisation, intensive care unit admission, ventilator use or mortality [35,36]. In this context, the present study found that asthma was not associated with an increased risk of ≥ severe COVID-19 or death during any wave, except for an increased risk of ≥ severe COVID-19 during the alpha-predominant wave. Interestingly, in this study, chronic pulmonary diseases other than asthma/COPD, were also associated with an increased risk of ≥ severe COVID-19 and death during any waves. Further investigation to identify which specific chronic pulmonary diseases are associated with these increased risks may yield clinically or epidemiologically meaningful information.

This study had several limitations. First, this study used claim data, so it did not include information on the body mass index, smoking history, blood or physiology test results or vaccines. In this study, we discussed changes in severe disease and mortality across the waves based on information on vaccination uptake in Japan (Supplementary Figure 1) as an alternative to an individual’s vaccination history. However, further studies that include information on individual vaccination histories are needed to better understand the impact of vaccination. Second, the definition of the predominant wave was based on screening data in Tokyo. Although Tokyo is the largest district in Japan, accounting for approximately 10% of the total Japanese population, it may not reflect the true epidemic situation in other areas. Third, our data did not include information about the omicron-predominant wave. By using the methodology of this study, we are planning to investigate the omicron-predominant wave and determine if the patient characteristics, outcomes and risk factors for severity/mortality change. The addition of information on omicron variants may provide more insight into the understanding of the different variants.

In conclusion, this large population-based study revealed that, from the early pandemic to the delta-predominant wave, the median age of the COVID-19-infected patients became younger, with decreasing severity/mortality, especially in the elderly. Although there were consistent risk factors for increased severity/mortality across the waves, some of those risks changed by wave. In the real world, there will be a significant change in viral mutations, the spread of vaccines and therapy in the future. Thus, continued analysis of big data from the past to the present and accumulation of evidence from each era will contribute to providing essential insights for the waves to come.

## Geolocation information

Asia.

## Supplementary Material

Supplemental Material

## Data Availability

The data that support the findings of this study are available from the Japan Ministry of Health, Labour and Welfare. Restrictions apply to the availability of these data, which were used under licence for this study.
