# Supplementary material for: Changes in the characteristics and outcomes of COVID-19 patients from the early pandemic to the delta variant epidemic: a nationwide population-based study
Source: Emerg Microbes Infect. 2022 Dec 20;12(1):2155250. doi: 10.1080/22221751.2022.2155250 (PMC9788709; doi:10.1080/22221751.2022.2155250)
Supplement: Supplemental Material [file TEMI_A_2155250_SM5333.docx]

**Supplementary material**

Supplementary Table 1. List of ICD-10 codes for comorbidities listed on the Charlson Comorbidity index

Supplementary Table 2. Incidences of ≥severe COVID-19 and of death in the overall population of this study by wave

Supplementary Table 3. Univariable logistic regression analyses for ≥severe COVID-19 and for death in all patients during the study period

Supplementary Table 4. Multivariable logistic regression analyses for ≥severe COVID-19 and for death in all patients during the study period

Supplementary Table 5. Multivariable logistic regression analyses for ≥severe COVID-19 by wave

Supplementary Table 6. Multivariable logistic regression analyses for death by wave

Supplementary Figure 1. Daily confirmed patients and cumulative vaccine uptake in Japan

Supplementary Figure 2. Multivariable logistic regression analyses for ≥severe and for death in the all patients during the study period

Supplementary Reference

**Supplementary Table 1.** **List of ICD-10 codes for comorbidities listed in the Charlson Comorbidity index**

| **Comorbidity** | **ICD-10 code** |
| --- | --- |
| Cerebrovascular disease | G45–46, I60–69, H340 |
| Any malignancy ^a^ | C00–26, C30–34, C37–41, C43, C45–58, C60–76, C81–85, C88, C90–97 |
| Dementia | F00–03, F51, G30, G311 |
| AIDS/HIV | B20–22, B24 |
| Myocardial infarction | I21–22, I252 |
| Renal disease | N18–19, I120, I131, N032–N037, N052–N057, N250, Z490–492, Z940, Z992 |
| Congestive heart failure | I43, I50, I099, I110, I130, I132, I255, I420, I425–429, P290 |
| Peripheral vascular disease | I70–71, I731, I738–739, I771, I790, I792, K551, K558–559, Z958–959 |
| Chronic pulmonary disease ^b^ |  |
| Asthma | J45–46 |
| Chronic obstructive pulmonary disease | J43–44 |
| Other chronic pulmonary disease | J40–42. J47, J60–67, I278–279, J684, J701, J703 |
| Rheumatic disease | M05–06, M32–34, M315, M 351, M353, M360 |
| Peptic ulcer disease | K25–28 |
| Liver disease | B18, I850, I859, I864, I982, K700–704, K709, K711, K713–715, K717, K721, K729, K760, K762–769, Z944 |
| Diabetes mellitus | E10–14 |
| Hemiplegia or paraplegia | G81–82, G041, G144, G801–802, G830–834, G839 |
| Metastatic solid tumours | C77–80 |

^a^ Any malignancy, including lymphoma and leukaemia, except malignant neoplasm of skin

^b^ Chronic pulmonary disease was divided into asthma, chronic obstructive pulmonary disease, and other chronic pulmonary disease

Coding algorithms for the Charlson Comorbidity Index were cited from Quan et al [1].

AIDS; acquired immunodeficiency syndrome, HIV; human immunodeficiency virus

[1] Quan H, Sundararajan V, Halfon P, et al. Coding algorithms for defining comorbidities in ICD-9-CM and ICD-10 administrative data. Med Care. 2005;43(11):1130-1139.

**Supplementary Table 2. Incidences of ≥severe COVID-19 and of death in the overall population of this study by wave**

|  | **Wave ^a^** | | | **Wild-type vs. Alpha ^b^** | | **Wild-type vs. Delta ^b^** | | **Alpha vs. Delta ^b^** | |
| --- | --- | --- | --- | --- | --- | --- | --- | --- | --- |
|  | **Wild-type**  **n = 365,929** | **Alpha**  **n = 196,957** | **Delta**  **n = 374,872** | **Risk difference**  **% (95% CI)** | **Risk ratio**  **(95% CI)** | **Risk difference**  **% (95% CI)** | **Risk ratio**  **(95% CI)** | **Risk difference**  **% (95% CI)** | **Risk ratio**  **(95% CI)** |
| ≥Severe COVID-19 ^c^ | 18,118  (5.0) | 8883  (4.5) | 5405  (1.4) | −0.4  (−0.6 to −0.3) | 0.91  (0.89 to 0.93) | −3.5  (−3.6 to −3.4) | 0.29  (0.28 to 0.30) | −3.1  (−3.2 to −3.0) | 0.32  (0.31 to 0.33) |
| Severe | 7340  (2.0) | 4643  (2.4) | 3719  (1.0) | 0.4  (0.3 to 0.4) | 1.18  (1.13 to 1.22) | −1.0  (−1.1 to −1.0) | 0.49  (0.48 to 0.51) | −1.4  (−1.4 to −1.3) | 0.42  (0.40 to 0.44) |
| Death | 10 778  (2.9) | 4240  (2.2) | 1686  (0.4) | −0.8  (−0.9 to −0.7) | 0.73  (0.71 to 0.76) | −2.5  (−2.6 to −2.4) | 0.15  (0.15 to 0.16) | −1.7  (−1.8 to −1.6) | 0.21  (0.20 to 0.22) |

^a^ Wild-type-predominant; January 1, 2020–April 18, 2021, alpha-predominant; April 19, 2021–July 18, 2021, delta-predominant; July 19, 2021–August 31, 2021

^b^ Earlier wave was used as the reference

^c^ Total number of patients with an outcome of severe COVID-19 or death

CI, confidence interval

**Supplementary Table 3. Univariable logistic regression analyses for ≥severe COVID-19 and for death in all patients during the study period**

|  | **≥severe ^a^** | |  | **Death** | |
| --- | --- | --- | --- | --- | --- |
|  | **OR (95% CI)** | ***P*-value** |  | **OR (95% CI)** | ***P*-value** |
| Age, (vs. 20–49), years |  |  |  |  |  |
| 50–64 | 6.90 (6.60−7.21) | <.001 |  | 10.06 (8.85−11.43) | <.001 |
| 65–79 | 21.2 (20.3−22.1) | <.001 |  | 86.5 (77.0−97.1) | <.001 |
| 80– | 42.8 (41.1−44.6) | <.001 |  | 336.5 (300.4−377.0) | <.001 |
| Male (vs female) | 1.58 (1.54−1.61) | <.001 |  | 1.14 (1.10−1.17) | <.001 |
| Wave, (vs Wild-type-predominant) |  |  |  |  |  |
| Alpha-predominant | 0.91 (0.88−0.93) | <.001 |  | 0.73 (0.70−0.75) | <.001 |
| Delta-predominant | 0.28 (0.27−0.29) | <.001 |  | 0.15 (0.14−0.16) | <.001 |
| Comorbidity, (vs absence) |  |  |  |  |  |
| Cerebrovascular disease | 5.56 (5.41−5.71) | <.001 |  | 8.22 (7.94−8.50) | <.001 |
| Any malignancy | 4.60 (4.46−4.74) | <.001 |  | 6.79 (6.54−7.05) | <.001 |
| Dementia | 7.19 (6.96−7.43) | <.001 |  | 14.6 (14.1−15.2) | <.001 |
| Myocardial infarction | 5.43 (5.12−5.75) | <.001 |  | 6.32 (5.89−6.79) | <.001 |
| Renal disease | 7.01 (6.76−7.28) | <.001 |  | 10.03 (9.60−10.47) | <.001 |
| Congestive heart failure | 6.76 (6.58−6.94) | <.001 |  | 10.5 (10.2−10.9) | <.001 |
| Peripheral vascular disease | 4.41 (4.26−4.56) | <.001 |  | 5.43 (5.20−5.67) | <.001 |
| Asthma | 1.70 (1.65−1.76) | <.001 |  | 1.71 (1.63−1.78) | <.001 |
| COPD | 6.41 (6.03−6.81) | <.001 |  | 7.84 (7.29−8.43) | <.001 |
| Other chronic pulmonary disease | 3.16 (3.06−3.26) | <.001 |  | 4.02 (3.86−4.18) | <.001 |
| Rheumatic disease | 3.11 (2.93−3.29) | <.001 |  | 3.69 (3.44−3.97) | <.001 |
| Peptic ulcer disease | 3.22 (3.12−3.31) | <.001 |  | 4.05 (3.90−4.20) | <.001 |
| Liver disease | 2.69 (2.61−2.76) | <.001 |  | 2.62 (2.52−2.72) | <.001 |
| Diabetes mellitus | 4.84 (4.72−4.95) | <.001 |  | 5.18 (5.02−5.35) | <.001 |
| Hemiplegia or paraplegia | 4.94 (4.51−5.42) | <.001 |  | 6.62 (5.95−7.36) | <.001 |
| Metastatic solid tumours | 6.57 (6.01−7.17) | <.001 |  | 11.2 (10.2−12.3) | <.001 |

CI, confidence interval; COPD, chronic obstructive pulmonary disease; OR, odds ratio

^a^ Based on the total number of patients with an outcome of severe COVID-19 or death

**Supplementary Table 4. Multivariable logistic regression analyses for ≥severe COVID-19 and for death in all patients during the study period**

|  | **≥severe ^a^** | |  | **Death** | |
| --- | --- | --- | --- | --- | --- |
|  | **OR (95% CI)** | ***P*-value** |  | **OR (95% CI)** | ***P*-value** |
| Age, (vs 20–49), years |  |  |  |  |  |
| 50–64 | 6.04 (5.78–6.32) | <.001 |  | 8.82 (7.76–10.0) | <.001 |
| 65–79 | 15.0 (14.4–15.7) | <.001 |  | 56.7 (50.4–63.8) | <.001 |
| 80– | 29.2 (27.8–30.6) | <.001 |  | 189.7 (168.6–213.4) | <.001 |
| Male (vs female) | 2.08 (2.03–2.13) | <.001 |  | 1.75 (1.69–1.81) | <.001 |
| Wave, (vs Wild-type-predominant) |  |  |  |  |  |
| Alpha-predominant | 1.16 (1.13–1.19) | <.001 |  | 0.99 (0.95–1.03) | .66 |
| Delta-predominant | 0.74 (0.71–0.76) | <.001 |  | 0.59 (0.56–0.62) | <.001 |
| Comorbidity, (vs absence) |  |  |  |  |  |
| Cerebrovascular disease | 1.06 (1.02–1.09) | .001 |  | 1.08 (1.04–1.13) | <.001 |
| Any malignancy | 1.07 (1.03–1.11) | <.001 |  | 1.31 (1.25–1.37) | <.001 |
| Dementia | 1.27 (1.23–1.32) | <.001 |  | 1.58 (1.52–1.65) | <.001 |
| Myocardial infarction | 1.01 (0.95–1.08) | .70 |  | 0.99 (0.92–1.07) | .78 |
| Renal disease | 1.48 (1.42–1.55) | <.001 |  | 1.67 (1.59–1.75) | <.001 |
| Congestive heart failure | 1.28 (1.24–1.33) | <.001 |  | 1.36 (1.31–1.42) | <.001 |
| Peripheral vascular disease | 1.02 (0.98–1.06) | .28 |  | 0.99 (0.95–1.04) | .76 |
| Asthma | 1.04 (1.01–1.08) | .03 |  | 0.92 (0.87–0.96) | <.001 |
| COPD | 1.19 (1.11–1.27) | <.001 |  | 1.21 (1.12–1.31) | <.001 |
| Other chronic pulmonary disease | 1.15 (1.11–1.19) | <.001 |  | 1.16 (1.11–1.22) | <.001 |
| Rheumatic disease | 1.19 (1.12–1.26) | <.001 |  | 1.20 (1.11–1.30) | <.001 |
| Peptic ulcer disease | 1.05 (1.01–1.08) | .006 |  | 1.07 (1.03–1.12) | .001 |
| Liver disease | 1.07 (1.04–1.11) | <.001 |  | 0.96 (0.92–1.00) | .04 |
| Diabetes mellitus | 1.28 (1.25–1.32) | <.001 |  | 1.06 (1.02–1.10) | .002 |
| Hemiplegia or paraplegia | 1.18 (1.07–1.30) | .001 |  | 1.23 (1.10–1.38) | <.001 |
| Metastatic solid tumours | 2.19 (1.99–2.42) | <.001 |  | 3.31 (2.97–3.69) | <.001 |

CI, confidence interval; COPD, chronic obstructive pulmonary disease; OR, odds ratio

Logistic regression model adjusted for age, sex, wave and comorbidities.

^a^ Based on the total number of patients with an outcome of severe COVID-19 or death

**Supplementary Table 5. Multivariable logistic regression analyses for ≥severe COVID-19 by wave**

|  | **Wild-type-predominant wave** | |  | **Alpha-predominant wave** | |  | **Delta-predominant wave** | |
| --- | --- | --- | --- | --- | --- | --- | --- | --- |
|  | **OR (95% CI)** | ***P*-value** |  | **OR (95% CI)** | ***P*-value** |  | **OR (95% CI)** | ***P*-value** |
| Age, (vs 20–49), years |  |  |  |  |  |  |  |  |
| 50–64 | 6.94 (6.40–7.53) | <.001 |  | 6.65 (6.10–7.25) | <.001 |  | 5.35 (5.00–5.74) | <.001 |
| 65–79 | 21.3 (19.8–23.0) | <.001 |  | 15.3 (14.0–16.6) | <.001 |  | 8.42 (7.66–9.25) | <.001 |
| 80– | 41.0 (37.9–44.3) | <.001 |  | 29.0 (26.4–31.8) | <.001 |  | 17.4 (15.6–19.4) | <.001 |
| Male (vs female) | 2.07 (2.00–2.14) | <.001 |  | 2.01 (1.91–2.10) | <.001 |  | 2.21 (2.08–2.35) | <.001 |
| Comorbidity, (vs absence) |  |  |  |  |  |  |  |  |
| Cerebrovascular disease | 1.07 (1.03–1.11) | .001 |  | 1.06 (1.00–1.13) | .07 |  | 0.95 (0.86–1.05) | .32 |
| Any malignancy | 1.11 (1.06–1.16) | <.001 |  | 1.00 (0.93–1.08) | .97 |  | 1.06 (0.95–1.18) | .38 |
| Dementia | 1.25 (1.19–1.30) | <.001 |  | 1.27 (1.17–1.37) | <.001 |  | 1.47 (1.29–1.67) | <.001 |
| Myocardial infarction | 1.03 (0.96–1.12) | .42 |  | 1.00 (0.88–1.13) | .98 |  | 0.91 (0.74–1.11) | .33 |
| Renal disease | 1.53 (1.45–1.61) | <.001 |  | 1.48 (1.36–1.60) | <.001 |  | 1.24 (1.09–1.42) | .001 |
| Congestive heart failure | 1.30 (1.25–1.35) | <.001 |  | 1.24 (1.17–1.32) | <.001 |  | 1.31 (1.19–1.44) | <.001 |
| Peripheral vascular disease | 1.03 (0.98–1.08) | .28 |  | 1.03 (0.95–1.11) | .50 |  | 0.98 (0.87–1.10) | .71 |
| Asthma | 1.03 (0.98–1.08) | .19 |  | 1.09 (1.01–1.17) | .02 |  | 1.03 (0.94–1.14) | .53 |
| COPD | 1.17 (1.07–1.27) | <.001 |  | 1.18 (1.03–1.35) | .02 |  | 1.34 (1.09–1.64) | .005 |
| Other chronic pulmonary disease | 1.16 (1.10–1.21) | <.001 |  | 1.14 (1.06–1.23) | <.001 |  | 1.12 (1.01–1.24) | .04 |
| Rheumatic disease | 1.24 (1.14–1.34) | <.001 |  | 1.07 (0.95–1.21) | .27 |  | 1.25 (1.06–1.48) | .01 |
| Peptic ulcer disease | 1.08 (1.03–1.12) | <.001 |  | 1.01 (0.94–1.07) | .85 |  | 1.01 (0.91–1.11) | .90 |
| Liver disease | 1.00 (0.96–1.04) | .97 |  | 1.13 (1.07–1.21) | <.001 |  | 1.29 (1.19–1.40) | <.001 |
| Diabetes mellitus | 1.27 (1.22–1.31) | <.001 |  | 1.22 (1.15–1.28) | <.001 |  | 1.53 (1.42–1.65) | <.001 |
| Hemiplegia or paraplegia | 1.13 (1.00–1.27) | .05 |  | 1.24 (1.02–1.51) | .03 |  | 1.34 (0.97–1.83) | .07 |
| Metastatic solid tumours | 2.32 (2.05–2.62) | <.001 |  | 1.78 (1.44–2.19) | <.001 |  | 2.52 (1.92–3.31) | <.001 |

CI, confidence interval; COPD, chronic obstructive pulmonary disease; OR, odds ratio

Logistic regression model adjusted for age, sex and comorbidities.

**Supplementary Table 6. Multivariable logistic regression analyses for death by wave**

|  | **Wild-type-predominant wave** | |  | **Alpha-predominant wave** | |  | **Delta-predominant wave** | |
| --- | --- | --- | --- | --- | --- | --- | --- | --- |
|  | **OR (95% CI)** | ***P*-value** |  | **OR (95% CI)** | ***P*-value** |  | **OR (95% CI)** | ***P*-value** |
| Age, (vs 20–49), years |  |  |  |  |  |  |  |  |
| 50–64 | 7.98 (6.57–9.69) | <.001 |  | 10.5 (7.96–13.9) | <.001 |  | 8.92 (7.17–11.1) | <.001 |
| 65–79 | 54.5 (45.7–65.0) | <.001 |  | 66.0 (51.0–85.4) | <.001 |  | 50.5 (40.8–62.6) | <.001 |
| 80– | 179.7 (150.8–214.3) | <.001 |  | 235.9 (182.3–305.3) | <.001 |  | 155.6 (125.1–193.6) | <.001 |
| Male (vs female) | 1.78 (1.70–1.86) | <.001 |  | 1.73 (1.61–1.85) | <.001 |  | 1.64 (1.47–1.82) | <.001 |
| Comorbidity, (vs absence) |  |  |  |  |  |  |  |  |
| Cerebrovascular disease | 1.11 (1.05–1.16) | <.001 |  | 1.06 (0.98–1.15) | .12 |  | 0.98 (0.86–1.12) | .73 |
| Any malignancy | 1.30 (1.23–1.37) | <.001 |  | 1.27 (1.17–1.39) | <.001 |  | 1.48 (1.29–1.69) | <.001 |
| Dementia | 1.56 (1.48–1.64) | <.001 |  | 1.61 (1.48–1.75) | <.001 |  | 1.72 (1.49–1.99) | <.001 |
| Myocardial infarction | 0.97 (0.88–1.07) | .56 |  | 0.98 (0.84–1.15) | .83 |  | 1.13 (0.88–1.45) | .35 |
| Renal disease | 1.68 (1.58–1.78) | <.001 |  | 1.76 (1.59–1.94) | <.001 |  | 1.36 (1.15–1.61) | <.001 |
| Congestive heart failure | 1.34 (1.28–1.41) | <.001 |  | 1.36 (1.26–1.47) | <.001 |  | 1.52 (1.34–1.73) | <.001 |
| Peripheral vascular disease | 0.98 (0.93–1.04) | .54 |  | 1.03 (0.94–1.13) | .55 |  | 0.97 (0.83–1.14) | .70 |
| Asthma | 0.92 (0.86–0.98) | .008 |  | 0.90 (0.81–1.00) | .05 |  | 0.93 (0.79–1.09) | .36 |
| COPD | 1.18 (1.07–1.30) | .001 |  | 1.27 (1.07–1.50) | .006 |  | 1.31 (1.01–1.69) | .04 |
| Other chronic lung disease | 1.16 (1.10–1.22) | <.001 |  | 1.15 (1.05–1.27) | .002 |  | 1.22 (1.05–1.42) | .009 |
| Rheumatic disease | 1.23 (1.12–1.36) | <.001 |  | 1.08 (0.92–1.27) | .34 |  | 1.31 (1.04–1.65) | .02 |
| Peptic ulcer disease | 1.07 (1.02–1.13) | .01 |  | 1.10 (1.01–1.19) | .03 |  | 1.03 (0.89–1.18) | .73 |
| Liver disease | 0.93 (0.88–0.99) | .01 |  | 0.94 (0.86–1.03) | .19 |  | 1.16 (1.01–1.32) | .03 |
| Diabetes mellitus | 1.08 (1.03–1.13) | <.001 |  | 0.99 (0.92–1.07) | .85 |  | 1.10 (0.98–1.24) | .11 |
| Hemiplegia or paraplegia | 1.17 (1.02–1.35) | .03 |  | 1.31 (1.03–1.66) | .03 |  | 1.55 (1.06–2.26) | .02 |
| Metastatic solid tumours | 3.42 (2.99–3.92) | <.001 |  | 2.67 (2.10–3.38) | <.001 |  | 3.98 (2.94–5.39) | <.001 |

CI, confidence interval; COPD, chronic obstructive pulmonary disease; OR, odds ratio

Logistic regression model adjusted for age, sex and comorbidities.

**Supplementary Figure 1. Daily confirmed patients and cumulative vaccine uptake in Japan**

**
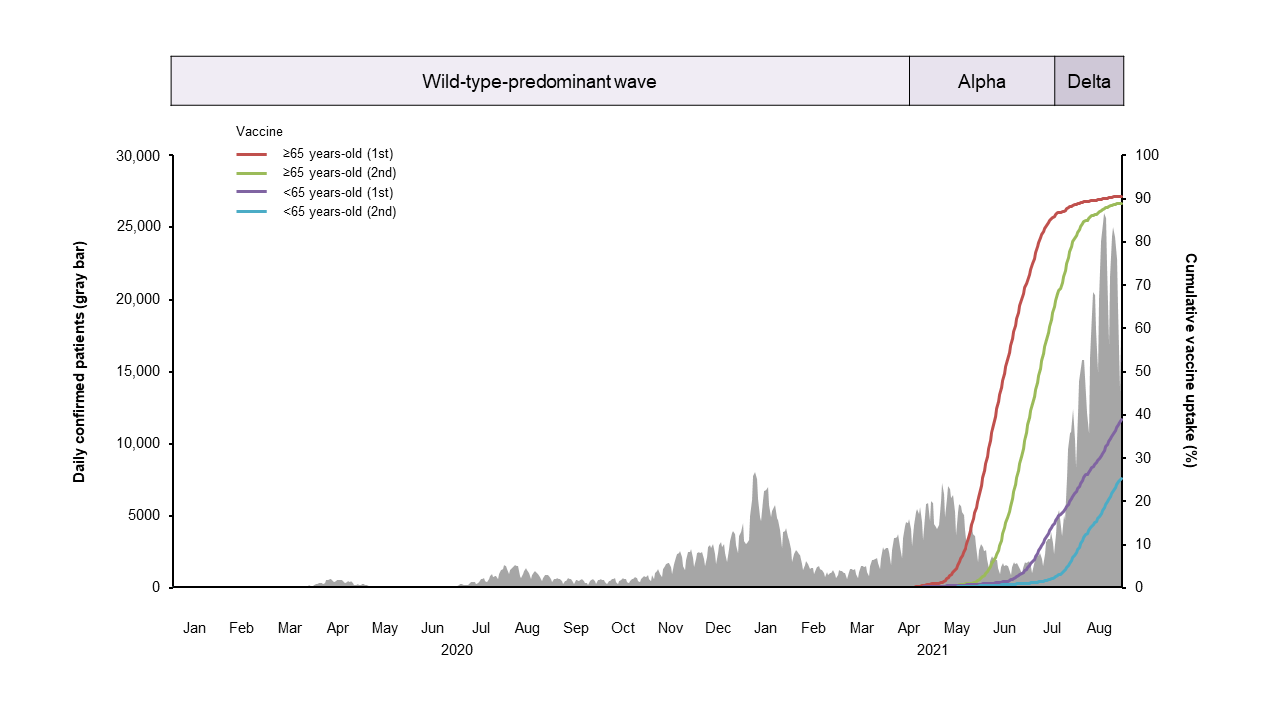
**

Number of daily confirmed patients with COVID-19 (grey bar)^E2^ and cumulative percentage of vaccines in patients aged ≥65 years who received 1 (red line) or 2 doses of vaccine (green line) and patients <65 years who received 1 (purple line) or 2 doses of vaccine (blue line).^E3^ Based on the trends of variants detected in Tokyo, Japan,^E4^ the period when the detection rate of a particular VOC exceeded 50% of the number of tests performed was defined as the predominant wave of that VOC, and the study period was divided as follows: 1) wild-type-predominant, from January 1, 2020 to April 18, 2021; 2) alpha-predominant, from April 19, 2021 to July 18, 2021; 3) delta-predominant, from July 19, 2021 to August 31, 2021 (the last date of the inclusion period of this study). In Japan, vaccination for public populations began on April 12, 2021, with priority given to the elderly and patients with some comorbidity. According to reports from the Japanese government,^E3^ at the peak of the alpha-predominant wave (May 8, 2021), 0.4% of the population < 65 years old and 1.4% of the population ≥ 65 years old had completed the first vaccination, and at the beginning of the delta-predominant wave (July 19, 2021), 15.0% of the population < 65 years old and 85.9% of the population ≥ 65 years old had completed the second vaccination. At the peak of the delta-predominant wave (August 20, 2021), 18.5% of the population < 65 and 87.7% of those ≥ 65 years old had completed their second vaccination. Briefly, during the alpha- and delta-predominant waves in Japan, vaccination became substantially more widespread, mainly among the elderly.

[2] Ministry of Health, Labor, and Welfare: Trend in the number of newly confirmed cases (daily). [cited 2022 May 2]. Available from: https://www.mhlw.go.jp/stf/covid-19/open-data_english.html.

[3] Digital Agency: The Vaccination Record System. Report in Japanese. [cited 2022 May 2]. Available from: https://info.vrs.digital.go.jp/dashboard/.

[4] Tokyo Metropolitan Government: Tokyo Metropolitan New Coronavirus Monitoring Conference and Analysis Materials. [cited 2022 Nov 8]. Available from: https://www.bousai.metro.tokyo.lg.jp/taisaku/saigai/1013388/

**Supplementary Figure 2. Multivariable logistic regression analyses for ≥severe COVID-19 and for death in all patients during the study period**

**
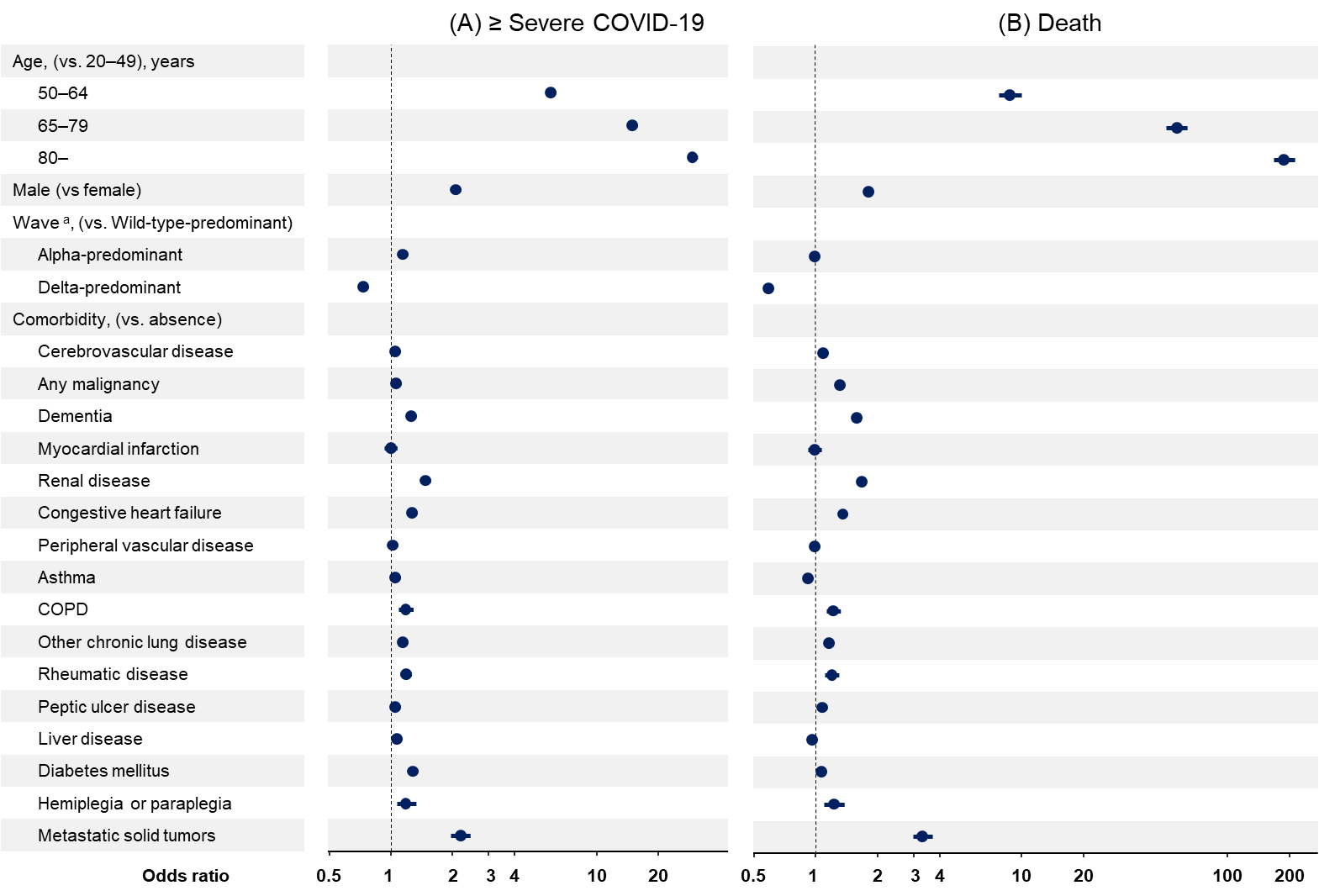
**

Adjusted odds ratio and 95% confidence intervals were plotted. The odd ratios were adjusted for age, sex, wave, and comorbidities.

^a^ Wild-type-predominant, January 1, 2020–April 18, 2021; alpha-predominant, April 19, 2021–July 18, 2021; delta-predominant, July 19, 2021–August 31, 2021.

COPD, chronic obstructive pulmonary disease

**Supplementary Reference**

1. Quan H, Sundararajan V, Halfon P, et al. Coding algorithms for defining comorbidities in ICD-9-CM and ICD-10 administrative data. Med Care. 2005;43(11):1130-1139.
2. Ministry of Health, Labour and Welfare: Trend in the number of newly confirmed cases (daily). [cited 2022 May 2]. Available from: https://www.mhlw.go.jp/stf/covid-19/open-data_english.html.
3. Digital Agency: The Vaccination Record System. [cited 2022 May 2]. Available from: https://info.vrs.digital.go.jp/dashboard/. Japanese.
4. Tokyo Metropolitan Government: Tokyo Metropolitan New Coronavirus Monitoring Conference and Analysis Materials. [cited 2022 Nov 8]. Available from: https://www.bousai.metro.tokyo.lg.jp/taisaku/saigai/1013388/
